# Association between liver enzymes and bone mineral density in Koreans: a cross-sectional study

**DOI:** 10.1186/s12891-018-2322-1

**Published:** 2018-11-24

**Authors:** Ho Jeong Do, Joon-Shik Shin, Jinho Lee, Yoon Jae Lee, Me-riong Kim, Dongwoo Nam, Eun-Jung Kim, Yeoncheol Park, Kristin Suhr, In-Hyuk Ha

**Affiliations:** 1 Jaseng Hospital of Korean Medicine, 536 Gangnam-daero, Gangnam-gu, Seoul, Republic of Korea; 2 Jaseng Spine and Joint Research Institute, Jaseng Medical Foundation, 538 Gangnam-daero, Gangnam-gu, Seoul, Republic of Korea; 3 Department of Acupuncture & Moxibustion, College of Korean Medicine, Kyung Hee University, 26 Kyungheedae-ro, Dongdaemun-gu, Seoul, Republic of Korea; 4 Department of Acupuncture & Moxibustion, College of Korean Medicine, Dongguk University, 123 Dongdae-ro, Gyeongju, Gyeongsangbuk-do Republic of Korea; 5 Department of Acupuncture & Moxibustion, Kyung Hee University Hospital at Gangdong, 892 Dongnam-ro, Gangdong-gu, Seoul, Republic of Korea; 6 Prevention Sciences, Rollins School of Public Health, Emory University, Atlanta, GA USA

**Keywords:** Osteoporosis, Bone density, Liver function tests, Cross-sectional studies, Health surveys

## Abstract

**Background:**

Osteoporosis is a major health concern for both men and women, and associated fractures incur substantial economic burden. While there are a multitude of studies on bone mineral density (BMD) and liver diseases, not many studies have assessed the association between liver enzyme levels and BMD in homogeneous populations.

**Methods:**

The current study investigated the association between serum liver enzyme levels and BMD at various sites in Koreans. Out of 21,517 surveyees of the 5th Korean National Health and Nutrition Examination Survey (2010–2012), 7160 participants’ data on BMD, serum liver enzymes, and full covariate data were included for cross-sectional analysis. BMD at the femoral neck, lumbar spine, entire femur, and whole body was assessed using dual energy X-ray absorptiometry (DEXA), and liver enzymes included aspartate aminotransferase (AST), alanine aminotransferase (ALT), and gamma(γ)-glutamyl transferase (GGT) levels. Differences in participant characteristics by BMD and liver enzyme levels were analyzed, and complex sample design regression analysis adjusted for multiple covariates was performed to assess the relationship between liver enzymes and BMD.

**Results:**

Negative associations were seen with GGT and BMD at all sites (*P* ≤ 0.02), ALT with lumbar spine (*P* = 0.0013), and AST with lumbar BMD (*P* = 0.0009). In particular, GGT presented strong negative associations with BMD in postmenopausal women and elder men.

**Conclusions:**

This study demonstrates a negative relationship between liver enzyme levels and BMD, and suggests that a significant association exists between osteoporosis/decreased BMD and liver disorders.

**Electronic supplementary material:**

The online version of this article (10.1186/s12891-018-2322-1) contains supplementary material, which is available to authorized users.

## Background

Osteoporosis is defined by the World Health Organization (WHO) as a disease whose main features are low bone mineral density (BMD) and deterioration of bone structure, leading to bone fragility and increased fracture susceptibility, especially in the hip, spine, and wrist regions [1]. Osteoporosis is a major health burden worldwide, and its significance is growing with the steady shift towards an aging society [2, 3]. A 2014 study estimated that the number of patients with osteoporosis or low bone mass (previously referred to as osteopenia) at the femoral neck or lumbar spine in adults aged 50 years or older would increase by 10.4 million from 2010 to 2020, and by 17.2 million from 2010 to 2030 as calculated by applying adjusted osteoporosis and low bone mass prevalence estimates from NHANES (2005–2010) data to the census population projections of 2020 and 2030 [4]. The situation in Korea is similar as evidenced by the steep increase in the number of patients aged 50 or older seeking medical services for osteoporosis, from 1.07 million in 2005, 1.2 million in 2006, and 1.33 million in 2007, to 1.46 million in 2008 in the Health Insurance Review and Assessment Service (HIRA) annual report [5].

Fractures from osteoporosis have been reported to incur considerable economic burden and increase morbidity [6]. Results from a study on the relationship between osteoporotic fractures and morbidity suggest an increase in risk of death in patients with incidental or pre-existing vertebral fractures or with other major osteoporotic fractures [7].

Early diagnosis and treatment of osteoporosis is of particular importance in the elderly as treatment and recovery from osteoporotic fractures are difficult [8]. Recently, multiple studies on risk factors influencing BMD were conducted, and a systematic review evaluating risk factors associated with BMD decrease in healthy men aged 50 or older revealed associations between bone loss and older age, smoking history, low weight, weight loss and physical/functional limitations. Other factors with a negative impact on BMD included low calcium intake, exercise amount, hyperthyroidism, hyperparathyroidism, and metabolic and endocrine disorders such as diabetes or chronic renal failure [9].

On the other hand, low BMD has been shown to be present in various hepatic disorders such as viral hepatitis, cholestatic liver disease, alcoholic cirrhosis, nonalcoholic fatty liver disease (NAFLD), hemochromatosis, and liver transplants [10].

Breitling [11] reported associations between liver enzymes such as aspartate aminotransferase (AST), alanine aminotransferase (ALT), and gamma(γ)-glutamyl transferase (GGT) with femoral neck BMD. AST, ALT, and GGT are widely used as markers of hepatic function; however, AST is less specific for liver function as it is also released from damaged cells in the heart, skeletal muscles, kidneys, pancreas, and lungs [12]. Meanwhile, GGT is involved in the extracellular catabolism of glutathione, an antioxidant, and is considered to be a marker of subclinical inflammation and oxidative stress [13, 14].

Although the study by Breitling suffers limitations as a cross-sectional study investigating BMD only in the femoral neck region, it also holds various strengths by considering for multiple covariates in a diverse population of non-Hispanic whites, African Americans, Mexican Americans, and others. The authors adopted the same study design to determine the association between liver enzyme levels and BMD in ethnic Koreans using the Korean National Health and Nutrition Examination Survey (KNHANES) data. The data was also compared to previous findings as prior studies were unable to assess BMD in Asians.

## Methods

### Study population and sampling

The dataset used in this study is from the 5th KNHANES (2010–2012). KNHANES is a national-level sample survey conducted by the Korean Ministry of Health and Welfare in citizens residing in South Korea but excludes Koreans in nursing homes, the military, and prison. KNHANES consists of a health-related survey, health examination, and nutritional assessment. KNHANES assessments were conducted by a team of specialized investigators in 4 regional areas (total 192 areas/year) every week (total 48 weeks/year). A mobile examination vehicle visited designated regional areas where health examinations and health-related surveys were conducted for 3 days. Within 1 week of the health examinations and surveys, a nutrition team visited the area and conducted nutritional assessment.

**Fig. 1:**
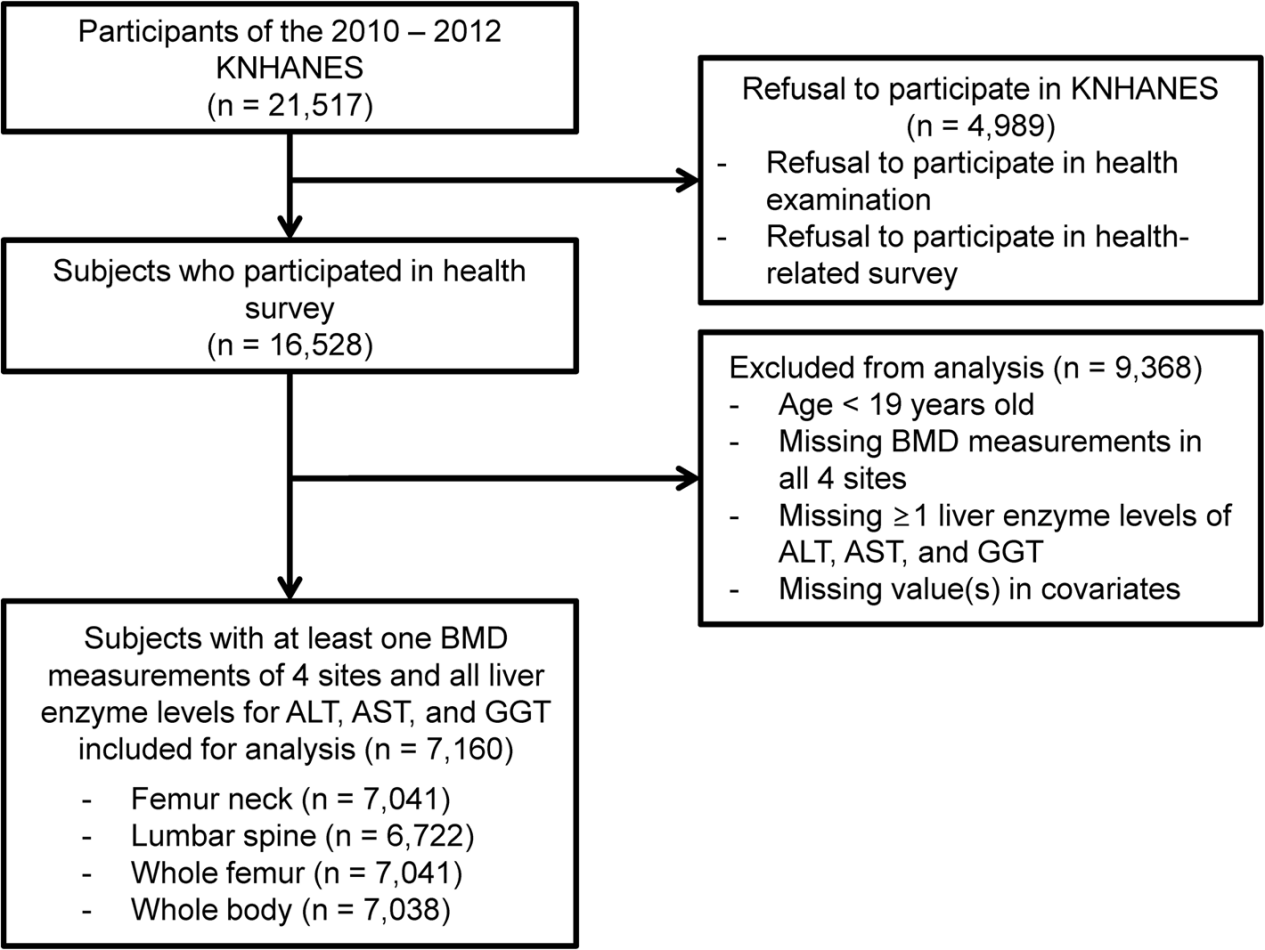
Flow diagram of selection of study subjects. KNHANES, Korea National Health and Nutrition Examination Survey; BMD, bone mineral density; ALT, alanine aminotransferase; AST, aspartate aminotransferase; GGT, γ-glutamyl transferase

KNHANES data can be downloaded from the official website (https://knhanes.cdc.go.kr/knhanes/index.do). This study used 2010 and 2011 data from the 5th KNHANES and included BMD results. The participation rate for health examinations and completion of health-related surveys was 76.8% (*n* = 16,528) out of 21,517 eligible participants. The BMD results for four regions (femoral neck, lumbar spine, entire femur, and whole body), and serum liver enzyme levels (AST, ALT, and GGT) of adult participants (age ≥ 19 years) were assessed, and the data of 7160 participants with results for all covariates were analyzed. Out of the 7160 participants, those with missing values for all four BMD sites, any liver enzymes, or covariates were excluded. Participants with spine, hip joint, or wrist fractures were additionally excluded from analysis (Fig. 1).

### BMD

BMD evaluations used femoral neck, lumbar spine, entire femur, and whole body BMD results from the KNHANES health examination. BMD was determined through dual energy X-ray absorptiometry (DEXA) and the measurement device used was Discovery W (Hologic; Marlborough, MA). Mobile examination centers were set up for BMD assessment and default examination sites were designated as the lumbar spine and left femur. If examination at the left femur was not possible due to lesions or disruptions such as surgery, fractures, abnormalities, or deformation, examination was conducted in the right femur. Follow-up measurements were not conducted.

### Liver enzyme levels

Serum AST, ALT, and GGT were quantified to assess liver enzyme levels. As liver enzyme levels show a nonlinear relationship with BMD, liver enzyme levels were converted to logarithmic values, and categorical variables were classified into quartiles.

### Covariates

Participant sociodemographic and economic characteristics investigated in this study included age, sex, and education level, and health-related characteristics included smoking and drinking status, moderate physical exercise, body mass index (BMI), diabetes mellitus history, and physician diagnosis of osteoporosis. Education levels were categorized into elementary school graduation or lower, middle school graduation, high school graduation, and college graduation or higher. Of health-related variables, smoking was categorized as follows: individuals with a lifetime smoking history of at least five packs of cigarettes (equivalent to 100 cigarettes) or more and were currently smoking were classified as ‘current smokers,’ those with a lifetime smoking history of five packs of cigarettes or more and were currently nonsmoking were designated as ‘previous smokers,’ and those with a smoking experience of less than five packs of cigarettes and were currently nonsmoking were designated ‘nonsmokers.’ Alcohol drinking was binary with individuals consuming alcohol once a month or more for the past year classified as ‘drinkers,’ and others as ‘nondrinkers.’ Regarding moderate physical exercise, participants who responded that they engaged in moderate physical exercise where breathing was slightly labored or more physically taxing than normal activities for one or more 30 min/sessions during the past week (e.g. leisurely swimming, doubles tennis, or volleyball) were defined as ‘regular exercisers’. BMI was analyzed as a continuous variable, and diabetes was classified into 3 groups: ‘diabetes’ when fasting blood glucose levels were ≥ 126 mg/dL or with intake of oral diabetes medication or insulin injection or with physician diagnosis of diabetes mellitus, ‘impaired fasting glucose’ when fasting plasma blood glucose levels were ≥ 100 mg/dL and ≤ 125 mg/dL, and ‘normal’ when fasting blood glucose levels were < 100 mg/dL. Physician diagnosis of osteoporosis was assessed through survey responses as to whether the participant had previously received diagnosis of osteoporosis from a physician.

Based on the study design by Breitling et al., this study examined associations between liver enzymes and BMD through adjusted analysis of risk factors affecting liver enzymes and BMD. Expert opinion of physicians and previous studies reporting risk factors relating to BMD and liver enzymes were considered in selection of appropriate covariates and models. Aging is the main factor for development of osteoporosis, and is more pronounced in women than in men due to hormonal changes [15]; therefore, age and sex were included as confounding variables. BMI and fasting blood glucose were considered based on reports accounting BMI and blood glucose as predictors of BMD [16], and smoking and limited physical activity were added as confounders on the basis of reports of associations with decreased BMD [9]. Additionally, a previous study reported that individuals with normal BMD showed higher education levels compared to those with low bone mass or osteoporosis [17], and level of education was also included as a confounding variable.

### Statistical methods

KNHANES is a national-level sample study and applies multistage cluster-sampling and weights. This study employs complex sample analysis using stratified, clustered, and weighted sampling as complex sample design factors, and all data analysis was conducted using statistical package SAS ver. 9.3 (SAS Institute Inc., Cary, NC). *P* < 0.05 was considered to be statistically significant. Descriptive statistics are presented by mean and 95% confidence intervals (CIs). Liver enzyme data showed right-skewed distribution, and data were converted to logarithmic values and calculated as geometric means. The difference in participant characteristics by BMD or liver enzyme levels were analyzed using one way analysis of variance (ANOVA). Whether liver enzyme levels exerted influence on BMD was assessed by conducting complex sample design regression analysis and adjusting for covariates. The results are presented using regression coefficients (β) and 95% CIs, and Taylor series linearization was used to calculate standard errors for estimates. In addition, subgroup analyses were performed by age and sex for pre−/post-menopause in women, and ages > and ≤ 41 years in men). Liver enzyme factors were applied as both categorical variables and logarithmic values to each model. The models were evaluated using adjusted R-square values, and the goodness of fit for each model was assessed for statistical significance using the F-test for the test of model effect.

## Results

### Sociodemographic characteristics and BMD

A total of 16,528 respondents participated in the health examination and survey out of 21,517 eligible participants from the 5th KNHANES. Data analysis was conducted in 7160 adults aged ≥19 years who had full data for BMD measurements at four regions, liver enzyme levels (AST, ALT, GGT), and covariates. The number of subjects differed slightly by BMD examination site with 7041 for the femoral neck, 6722 for the lumbar spine, 7041 for the femur, and 7038 for whole body BMD.

Table 1 presents the association between participant characteristics and BMD values at the four sites. BMD was lower in women compared to men. Participants with higher education levels displayed higher BMD, as did ex-smokers and current smokers compared to nonsmokers. Subjects with alcohol intake at least once a month showed higher BMD scores than those with lower alcohol consumption (*p* < .0001). Meanwhile, respondents reporting diagnosis of osteoporosis were shown to have lower BMD compared to those reporting no osteoporosis (*p* < .0001). BMD by moderate physical activity differed by BMD examination site, with BMD for the femoral neck and entire femur showing significantly higher association with moderate physical activity, but nonsignificant associations with other regions. Also, participants with normal or impaired fasting glucose levels displayed higher BMD than those with diabetes, and especially at the femoral neck. However, BMD for the entire femur did not show significant difference in association with diabetes.Liver enzyme levels and covariates.

**Table 1:** Bone mineral density at various measurement sites by demographic characteristics

	Femur neck BMD		Lumbar spine BMD		Whole femur BMD		Whole body BMD	
	n	Mean (SD)	n	Mean (SD)	n	Mean (SD)	n	Mean (SD)
Total	7041		6722		7041		7038	
Age (years)		48.38 (15.77)		47.78 (15.44)		48.38 (15.77)		48.21 (15.71)
19~ 29	876	0.85	846	0.99	876	0.95	886	1.16
30~ 44	2261	0.80	2236	0.98	2261	0.94	2279	1.16
45~ 59	1985	0.76	1919	0.94	1985	0.91	1982	1.14
60~ 74	1567	0.67	1430	0.87	1567	0.83	1550	1.07
≥ 75	352	0.58	291	0.81	352	0.72	341	1.00
*p-value*		<.0001		<.0001		<.0001		<.0001
Sex								
Male	3060	0.83	2935	0.97	3060	0.97	3035	1.18
Female	3981	0.72	3787	0.93	3981	0.85	4003	1.09
*p-value*		<.0001		<.0001		<.0001		<.0001
Education level								
≤ Elementary school graduation	1611	0.66	1437	0.85	1611	0.81	1578	1.05
Middle school graduation	757	0.74	720	0.93	757	0.90	759	1.12
High school graduation	2405	0.81	2332	0.98	2405	0.94	2418	1.16
≥ College graduation	2268	0.80	2233	0.98	2268	0.93	2283	1.16
*p-value*		<.0001		<.0001		<.0001		<.0001
Smoking status								
Nonsmoker	4271	0.75	4074	0.94	4271	0.88	4292	1.11
Previous smoker	1277	0.79	1215	0.96	1277	0.94	1267	1.16
Current smoker	1493	0.82	1433	0.97	1493	0.96	1479	1.18
*p-value*		<.0001		<.0001		<.0001		<.0001
Body mass index (kg/m^2^)		23.62 (3.37)		23.61 (3.34)		23.62 (3.37)		23.60 (3.35)
Drinking status								
No	3166	0.73	2989	0.93	3166	0.87	3160	1.10
Yes	3875	0.80	3733	0.97	3875	0.94	3878	1.16
*p-value*		<.0001		<.0001		<.0001		<.0001
Moderate physical exercise								
No	6281	0.77	5994	0.95	6281	0.91	6284	1.14
Yes	760	0.79	728	0.96	760	0.93	754	1.13
*p-value*		0.01		0.19		0.00		0.37
History of osteoporosis physician diagnosis								
No	6603	0.78	6318	0.96	6603	0.92	6608	1.14
Yes	336	0.60	305	0.78	336	0.74	329	0.99
*p-value*		<.0001		<.0001		<.0001		<.0001
History of diabetes								
Normal	5158	0.78	4935	0.96	5158	0.91	5165	1.14
IFG	1222	0.76	1170	0.95	1222	0.92	1214	1.14
DM	661	0.73	617	0.94	661	0.90	659	1.12
*p-value*		<.0001		0.05		0.08		0.02

Continuous variables are presented as mean ± SD (standard deviation) *p*-value calculated using ANOVA test

BMD, bone mineral density; IFG, impaired fasting glucose; DM, diabetes mellitus

Table 2 shows that nonsmokers presented with lower levels in all liver enzymes (*p* < .0001). In addition, among alcohol drinkers, those with impaired fasting glucose or diabetes had higher liver enzyme levels than those without (*p* < .0001). Moderate level physical activity did not exhibit a statistically significant relationship in association with liver enzymes. Meanwhile, higher AST was associated with lower education levels (*p* < .0001), and higher ALT and GGT were similarly related to education levels of middle school graduation or lower. Previous osteoporosis diagnosis was associated with higher AST and lower GGT compared to those without a history of osteoporosis, while ALT did not show a statistically significant relationship.

**Table 2:** Liver function test results by demographic characteristics

	n	AST	ALT	GGT
		mean^a^	mean^a^	mean^a^
Total	7160			
Sex				
Male	3093	22.60	22.83	34.22
Female	4067	18.66	14.92	17.28
*p-value*		<.0001	<.0001	<.0001
Education level				
≤ Elementary school graduation	1622	22.51	18.72	25.11
Middle school graduation	767	21.87	19.73	27.70
High school graduation	2450	20.09	18.08	24.00
≥ College graduation	2321	19.75	18.37	23.36
*p-value*		<.0001	0.01	<.0001
Smoking status				
Nonsmoker	4356	19.15	16.01	18.55
Previous smoker	1292	22.58	22.07	30.92
Current smoker	1512	22.33	22.07	36.55
*p-value*		<.0001	<.0001	<.0001
Drinking status				
No	3227	19.71	17.11	18.78
Yes	3933	21.09	19.39	28.83
*p-value*		<.0001	<.0001	<.0001
Moderate physical exercise				
No	6396	20.49	18.45	24.27
Yes	764	20.83	18.39	24.54
*p-value*		0.33	0.87	0.75
History of osteoporosis physician diagnosis				
No	6720	20.47	18.43	24.41
Yes	338	21.28	17.72	20.36
*p-value*		0.04	0.12	<.0001
History of diabetes				
Normal	5247	19.71	17.14	21.78
IFG	1241	23.18	22.87	33.93
DM	672	23.87	24.36	35.83
*p-value*		<.0001	<.0001	<.0001

^a^ Geometric mean*. p*-value calculated using ANOVA test

AST, aspartate aminotransferase; ALT, alanine aminotransferase; GGT, γ-glutamyl transferase; IFG, impaired fasting glucose; DM, diabetes mellitus

### Liver enzyme levels and BMD

Table 3 shows the multiple regression analysis results for the association between liver enzyme levels and BMD values for the femoral neck and lumbar area adjusting for covariates (data for the association between liver enzyme levels and BMD values for the entire femur and whole body are presented as Additional file 1: Table S1). Also, change in BMD considering for the right-skewed distribution of liver enzyme levels through logarithm conversion was investigated to assess the relationship between liver enzyme levels and BMD. The median values and interquartile ranges (IQRs) for liver enzyme levels were 19 U/L (16-23 U/L) for AST, 16 U/L (12-23 U/L) for ALT, and 19 U/L (13-32 U/L) for GGT.

**Table 3:** Association between liver function tests and femur neck and lumbar bone mineral density as assessed using regression analysis models^a^

	Model 1			Model 2			Fully adjusted model		
	β	95% CI	*p*-value	β	95% CI	*p*-value	β	95% CI	*p*-value
Femur neck									
AST (IU/L)									
AST > 23	0.0177	(0.0079,0.0276)	0.0005	−0.0003	(− 0.01,0.0095)	0.9559	− 0.0003	(− 0.0102,0.0096)	0.9492
19 > AST ≤ 23	0.0100	(0.0004,0.0196)	0.0419	−0.0009	(− 0.01,0.0083)	0.8493	− 0.0008	(− 0.0099,0.0083)	0.8572
16 < AST ≤ 19	0.0015	(− 0.0088,0.0119)	0.7732	− 0.0024	(− 0.012,0.0072)	0.6253	− 0.0023	(− 0.0118,0.0072)	0.631
AST ≤ 16	Ref.			Ref.			Ref.		
Adjusted R-Square	0.3660			0.4313			0.4381		
per log unit			0.0002			0.7253			0.7521
ALT (IU/L)									
ALT > 23	0.0384	(0.0293,0.0475)	<.0001	−0.0027	(−0.0131,0.0076)	0.6005	−0.0016	(−0.012,0.0089)	0.7688
16 < ALT ≤23	0.0231	(0.0132,0.033)	<.0001	−0.0009	(−0.0103,0.0085)	0.8486	−0.0002	(− 0.0096,0.0092)	0.9659
12 < ALT ≤16	0.0165	(0.0082,0.0247)	0.0001	0.0019	(−0.006,0.0099)	0.6302	0.0019	(− 0.006,0.0098)	0.6345
ALT ≤12	Ref.			Ref.			Ref.		
Adjusted R-Square	0.3721			0.4314			0.4381		
per log unit			<.0001			0.1635			0.2921
GGT (IU/L)									
GGT > 32	0.0208	(0.0107,0.0308)	<.0001	−0.0164	(− 0.0267,-0.0061)	0.0019	−0.0196	(− 0.0305,-0.0088)	0.0005
19 < GGT ≤32	0.0196	(0.01,0.0293)	<.0001	−0.0066	(−0.0162,0.0029)	0.1721	−0.0082	(− 0.0178,0.0015)	0.0958
13 < GGT ≤19	0.0076	(−0.002,0.0172)	0.1183	−0.0042	(−0.0133,0.0049)	0.3661	−0.0048	(− 0.0137,0.004)	0.2824
GGT ≤13	Ref.			Ref.			Ref.		
Adjusted R-Square	0.3663			0.4325			0.4398		
per log unit			0.0028			0.0015			0.0004
Lumbar spine									
AST (IU/L)									
AST > 23	−0.0042	(− 0.0158,0.0074)	0.4788	− 0.0204	(− 0.0322,-0.0086)	0.0007	− 0.0187	(− 0.0306,-0.0069)	0.002
19 > AST ≤ 23	− 0.0113	(− 0.0228,0.0001)	0.0518	− 0.0204	(− 0.0315,-0.0094)	0.0003	− 0.0190	(− 0.0297,-0.0083)	0.0005
16 < AST ≤ 19	− 0.0135	(− 0.0244,-0.0026)	0.0156	− 0.0166	(− 0.0271,-0.0061)	0.0021	− 0.0156	(− 0.0258,-0.0053)	0.0031
AST ≤ 16	Ref.			Ref.			Ref.		
Adjusted R-Square	0.1185			0.1745			0.2022		
per log unit			0.5807			0.0004			0.0009
ALT (IU/L)									
ALT > 23	0.0213	(0.0093,0.0332)	0.0005	−0.0187	(− 0.0319,-0.0054)	0.0061	−0.0186	(− 0.032,-0.0052)	0.0068
16 < ALT ≤23	0.0098	(−0.0005,0.0201)	0.0633	−0.0133	(− 0.0239,-0.0027)	0.0138	− 0.0100	(− 0.0208,0.0008)	0.0682
12 < ALT ≤16	−0.0007	(− 0.0102,0.0089)	0.8909	−0.0152	(− 0.0243,-0.006)	0.0012	− 0.0129	(− 0.022,-0.0038)	0.0057
ALT ≤12	Ref.			Ref.			Ref.		
Adjusted R-Square	0.1206			0.1734			0.2014		
per log unit			0.0014			0.0013			0.0013
GGT (IU/L)									
GGT > 32	0.0091	(−0.0044,0.0225)	0.1856	−0.0260	(− 0.0397,-0.0122)	0.0003	− 0.0276	(− 0.0422,-0.013)	0.0002
19 < GGT ≤32	0.0085	(−0.0032,0.0203)	0.1549	−0.0156	(− 0.0273,-0.0039)	0.0091	− 0.0145	(− 0.0262,-0.0028)	0.0154
13 < GGT ≤19	− 0.0075	(− 0.0193,0.0042)	0.207	− 0.0187	(− 0.0297,-0.0078)	0.0009	− 0.0157	(− 0.0264,-0.005)	0.0043
GGT ≤13	Ref.			Ref.			Ref.		
Adjusted R-Square	0.1192			0.1745			0.2025		
per log unit			0.2331			0.004			0.0013

^a^ Model 1: adjusted for age, and sex;

Model 2: adjusted for age, sex, and body mass index (BMI);

Fully adjusted model: adjusted for age, sex, alcohol use, BMI, smoking status, diabetes, physical activity, and education

Age and BMI are adjusted as continuous variables

AST, aspartate aminotransferase; ALT, alanine aminotransferase; GGT, γ-glutamyl transferase

In the age- and sex-adjusted model (Model 1), ALT displayed positive associations with BMD at all sites, and GGT showed positive associations with BMD at two areas excluding lumbar and whole body BMD (see Additional file 1: Table S1). AST showed positive associations with the femoral neck and whole femur BMD (Additional file 1: Table S1).

When Model 1 was further adjusted for alcohol use, no significant differences between the two models emerged (data not shown). However, when Model 1 was further adjusted for BMI (Model 2), a statistically significant negative association between GGT and BMD in the femur neck was observed, and negative associations between all liver enzyme levels (i.e., AST, ALT, and GGT) and BMD in the lumbar spine were also statistically significant.

When Model 1 was further adjusted for BMI and alcohol intake to yield Model 2, Model 2 displayed negative associations where BMD values at all regions increased with decreasing GGT values in contrast to Model 1, and AST and ALT values also exhibited similar relationships with BMD at the lumbar spine. In addition, ALT demonstrated a nonlinear, non-logarithmic association with whole body BMD by quartile interval (Additional file 1: Table S1).

The fully adjusted model was adjusted for smoking, diabetes, physical activity, and education level in addition to Model 2 factors. The fully adjusted model maintained statistical significance as in Model 2.

In Table 3, the fully adjusted model was confirmed to have the best goodness of fit followed by Model 2 and Model 1, as assessed by adjusted R-square values.

Table 4 shows stratification results by sex. Subjects included for analysis were the participants included in the fully adjusted model as shown in Table 3. The BMD examination sites were designated as the femoral neck and lumbar spine as recommended by the National Osteoporosis Foundation for defining osteoporosis or low bone mass [18].

**Table 4:** Subgroup analysis stratified by sex using multivariable-adjusted regression model^a^

	Male			Female		
	β	95% CI	p-value	β	95% CI	p-value
Femur neck						
AST						
AST > 23	− 0.0026	(− 0.0175,0.0122)	0.7281	0.0070	(−0.006,0.02)	0.2876
19 < AST ≤23	0.0023	(−0.0126,0.0173)	0.7596	−0.0021	(−0.0123,0.0081)	0.6879
16 < AST ≤19	−0.0018	(−0.0187,0.0151)	0.8328	−0.0005	(− 0.0109,0.0098)	0.9203
AST ≤16	Ref.			Ref.		
per log unit			0.7181			0.8203
ALT						
ALT > 23	0.0024	(−0.016,0.0207)	0.7997	0.0013	(−0.0121,0.0148)	0.8480
16 < ALT ≤23	0.0032	(−0.0148,0.0211)	0.7288	0.0010	(−0.0099,0.012)	0.8501
12 < ALT ≤16	0.0082	(−0.0098,0.0261)	0.3719	0.0011	(−0.0078,0.0099)	0.8144
ALT ≤12	Ref.			Ref.		
per log unit			0.2549			0.5154
GGT						
GGT > 32	−0.0153	(−0.0413,0.0107)	0.2485	−0.0138	(−0.0278,0.0003)	0.0549
19 < GGT ≤32	−0.0001	(−0.0269,0.0267)	0.9941	−0.0102	(− 0.0219,0.0015)	0.0862
13 < GGT ≤19	0.0011	(−0.0258,0.028)	0.9363	−0.0041	(−0.0124,0.0041)	0.3248
GGT ≤13	Ref.			Ref.		
per log unit			0.0018			0.1700
Lumbar spine						
AST						
AST > 23	−0.0163	(−0.0351,0.0024)	0.0878	−0.0090	(−0.024,0.0061)	0.2426
19 < AST ≤23	−0.0115	(−0.0296,0.0066)	0.2125	−0.0136	(− 0.0271,-0.0002)	0.0472
16 < AST ≤19	−0.0162	(− 0.035,0.0027)	0.0922	− 0.0020	(− 0.0137,0.0097)	0.7346
AST ≤16	Ref.			Ref.		
per log unit			0.0562			0.0431
ALT						
ALT > 23	−0.0074	(−0.0297,0.0149)	0.5139	−0.0048	(− 0.0228,0.0131)	0.5952
16 < ALT ≤23	0.0015	(−0.0195,0.0225)	0.8887	−0.0039	(−0.0167,0.0088)	0.5437
12 < ALT ≤16	−0.0046	(−0.0259,0.0167)	0.6731	−0.0045	(− 0.0161,0.0071)	0.4461
ALT ≤12	Ref.			Ref.		
per log unit			0.1372			0.4714
GGT						
GGT > 32	−0.0158	(−0.0393,0.0077)	0.1874	−0.0109	(−0.0315,0.0096)	0.2957
19 < GGT ≤32	0.0036	(−0.0208,0.028)	0.7702	−0.0095	(−0.0229,0.0039)	0.1648
13 < GGT ≤19	0.0018	(−0.0256,0.0291)	0.8981	−0.0102	(−0.0209,0.0005)	0.0623
GGT ≤13	Ref.			Ref.		
per log unit			0.0008			0.4578

^a^Adjusted for age, body mass index (BMI), alcohol use, diabetes, physical activity, education, and smoking status

AST, aspartate aminotransferase; ALT, alanine aminotransferase; GGT, γ-glutamyl transferase

GGT demonstrated negative associations with the femoral neck and lumbar BMD in men, and statistical significance was especially pronounced in older age groups (> 41 years) (Additional file 1: Table S3). While statistical significance for liver enzymes and femoral neck and lumbar BMD was not observed in women of all ages, GGT displayed a negative association with femoral neck BMD in postmenopausal women (Additional file 1: Table S3)

## Discussion

This study investigated the association between liver enzyme levels and BMD in Korean adults aged 19 or older using KNHANES data. ALT generally displayed a negative relationship with the lumbar spine and whole body BMD, and while AST showed a negative relationship with lumbar BMD, associations with BMD at other regions were nonsignificant. Meanwhile, GGT exhibited relatively clear negative associations with BMD at multiple sites. The association between GGT and femoral neck and lumbar BMD was especially pronounced in older men compared to their younger counterparts, and was similar to the relationship with femoral neck BMD in postmenopausal women compared to premenopausal women. Out of the four BMD examination regions (femoral neck, entire femur, lumbar spine, and whole body), liver enzyme levels generally showed significant associations with the lumbar spine, and significance was maintained after adjusting for various confounding variables.

### Effects of age and sex on liver enzyme levels and BMD

Since age and sex are the predominant predictors for BMD, models from Table 3 were further adjusted for age-sex interactions, and their statistical significance was assessed (Additional file 1: Table S2). Further adjustment tended to weaken associations between AST, ALT and BMD; however, it did not affect the negative relationship between GGT and BMD. Since BMD in women is closely associated with menopause, other factors are more likely than liver enzyme levels to influence BMD by age. Therefore, further age-stratified analyses (i.e., pre/postmenopause in women and ages > and ≤ 41 in men) were performed (Additional file 1: Table S3). GGT tended to show negative associations with BMD of the femur neck especially in postmenopausal women, but this trend was not observed in men. Further studies should be conducted to explain this difference.

### Effects of BMI as a major variable

BMI was shown to have a large effect on the association between BMD and liver enzyme levels in this study. Weight is closely associated with bone mass, and it can be carefully conjectured that bone mass is low and bone loss increases in postmenopausal women with low BMI. On the other hand, BMD tends to be high in obese women [19], and the BMI of osteoporotic patients was 0.763 times that of their non-osteoporotic counterparts [16]. This may be because as weight increases, muscle load and mechanical stress also increases, leading to better maintenance of bone mass [20]. Moreover, body fat has been purported to affect BMD through regulation of the levels of circulating sex hormones such as estradiol and sex hormone-binding globulins [21, 22].

Obesity, indicated as an increase in BMI, is closely associated with development of type 2 diabetes mellitus, which is likely to increase risk of chronic liver diseases and cancers such as hepatocellular carcinoma [23, 24]. This may be because increased fasting glucose levels and insulin resistance, which are key factors in development of type 2 diabetes mellitus, hold similar properties and implications as elevated GGT activity. In addition, BMI and fasting blood glucose showed stronger associations with GGT compared to ALT, and high insulin resistance was associated with elevated ALT activity in individuals with hepatic diseases [25–27].

### Liver diseases and BMD

Various studies have demonstrated associations between hepatic disease and osteoporosis or decreased bone mass. Osteoporosis prevalence was reported to vary from 11 to 58% in chronic liver disease patients and liver transplant recipients, and low bone mass has been observed in various liver disorders including viral hepatitis, cholestatic liver disease, alcoholic cirrhosis, NAFLD, hemochromatosis, and liver transplants [10]. In a previous study by Breitling using a comparable study design and model, a weak negative association was perceived between serum GGT and femoral neck BMD, whereas AST failed to reach statistical significance in associations with femoral neck BMD, and ALT showed a limited association with femoral neck BMD [11]. Compared to the previous study, this study revealed stronger negative relationships between GGT and ALT, and BMD, and did not show the U-shaped relationships with BMD commonly seen at lower values.

### Effects of GGT on BMD

GGT exhibited a relatively strong negative statistical significance with BMD at various sites in the current study, and the relationship between GGT and BMD has also been examined in numerous other studies [28, 29]. A cross-sectional study conducted in 462 Korean adults demonstrated a negative relationship between serum GGT and BMD [30], and a large prospective cohort study on 16,036 Korean men aged ≥50 years found that elevated serum GGT levels at baseline were significantly associated with an increased incident of osteoporotic fractures over an average 3-year follow-up period, indicating that higher serum GGT levels may act as an independent risk factor for development of incidental fractures at osteoporosis-related sites in men [31]. In addition, Niida et al. suggested that GGT stimulates the receptor activator of nuclear factor-kappa ß ligand expression and induces osteoclast formation [32], and GGT overexpression has been linked with accelerated bone resorption and osteoporosis development in transgenic mice [33], lending weight to the hypothesis that GGT may play a direct role in the pathogenesis of metabolic bone disease. Moreover, GGT is known to be involved with extracellular catabolism of the antioxidant glutathione [34], and has also been positively associated with inflammation markers such as C-reactive protein and fibrinogen [35]. Several in vitro studies have shown that oxidative stress upregulates osteoclast activity [36] and inhibits osteoblast differentiation [37]. Oxidative stress and inflammation induced by high GGT has been inferred to contribute to osteoporotic fracture incidence. However, although GGT increases in proportion to alkaline phosphatase (ALP) in hepatic disorders and is the most sensitive marker for biliary disease, GGT increase is nonspecific and associated not only with pancreatic, cardiac, renal, and pulmonary diseases but also diabetes and alcoholism [38].

### ALT and BMD

In the current study, ALT levels displayed statistically significant negative associations with the lumbar spine and whole body BMD upon adjustment for various confounding variables. Breitling [11] noted a U-shaped association between ALT and femoral neck BMD, and similar U-shaped associations were observed for ALT and mortality, suggesting that very low ALT levels indicate decrepitude. These results, however, are highly disparate with current study findings where the lowest ALT quartile was linked with high bone mass. These findings may be a result of the difference in BMD measurement region, or alternately be a characteristic of ethnic divergence. While 7.4% of the Korean population displayed serum ALT elevation [39], 7.9% of the U.S. population presented aminotransferase elevation of whom 32% showed ALT increase, 25.6% AST increase, and 42.4% increase in both ALT and AST [40], roughly amounting to a 5.5% ALT increase in the U.S. population. It therefore seems necessary to factor in the influence of ethnic origin in the association between ALT and BMD. Also, a 2014 study conducted in Koreans asserted that NAFLD was the first disorder to be considered in Koreans showing elevated ALT [39], while other recent studies have reported associations between NAFLD and decreased bone mass [41–43]. As the KNHANES data used in the current study did not include survey items or examinations identifying NAFLD prevalence, stratified analysis or variable adjustment for NAFLD could not performed, indicating the need for future large-scale, population-based studies including NAFLD data to determine the potential effect of NAFLD on the association between ALT and BMD.

### Limitations

The largest limitation of the current study is that only associations between variables, as opposed to causal or sequential relationships, could be identified due to its cross-sectional design. Other limitations include using quartiles for serum AST, ALT, GGT - though convenient for assessing potential associations between liver enzyme levels and BMD - may be regarded to be a restriction in clinical application as the quartile ranges are discordant with normal ranges. Also, this study did not investigate or consider mineral intake, calcium consumption through foods, or serum vitamin D, although previous studies have stated that no significant change in the relationship between liver enzymes and BMD was noticed following adjustment for these variables [11]. While this study included investigation of the impact of BMI on liver enzymes and BMD, other longitudinal studies have reported relationships not only between low body weight but also weight loss and low BMD [44–49]. Although the KNHANES survey contained an item inquiring about weight loss over the previous year, the data was not included as BMD results before and after weight loss could not be utilized due to the cross-sectional design of the current study. In addition, this study failed to consider all factors previously reported to influence liver enzyme elevation. For instance, prior studies have suggested that muscle injury or musculoskeletal diseases may increase liver enzyme levels, and though isolated aminotransferase increase is generally regarded to be benign, it has been linked with liver cirrhosis or fibrosis following unexplained elevation in some patients [50–53].

### Strengths

The most distinctive strength of this study is that the investigation and analyses were conducted on a large-scale sample representative of the South Korean population. It is also necessary to consider for the effects of geographical and environmental factors as well as ethnicity in investigating associations between liver enzymes and BMD. According to previous studies, low bone mass and osteoporosis of the femoral head in men was most prevalent in the U.S. followed by Korea and Saudi Arabia [54, 55], and difference in osteoporosis prevalence by ethnicity following BMD measurement sites has also been reported [54, 56]. In a study conducted in Americans aged 17 years or older, increased serum aminotransferase (ALT or AST) levels were noted in 14.9% of Mexican Americans, 8.1% of non-Hispanic blacks, and 7.1% of non-Hispanic whites [40]. Difference in prevalence of hepatic steatosis was observed by ethnicity and sex. Specifically, hepatic steatosis prevalence was 45% in Hispanics, 33% in Caucasians, and 24% in non-Hispanic blacks, and 42 and 24% in Caucasian men and women, respectively [57]. The authors would like to draw attention to the fact that this study may be considered to hold higher internal validity compared with previous studies as it was implemented using KNHANES data collected from a relatively homogeneous population of Koreans. An added strength is that this study analyzed a wide range of BMD measurement areas covering the femoral neck, lumbar spine, whole femur, and whole body. The National Osteoporosis Foundation issued recommendations that both the femoral neck and lumbar spine BMD should be taken into account in defining osteoporosis and low BMD [18], while the WHO has stated that the femoral neck is the only region that should be used in osteoporosis prevalence estimations at population levels [58, 59]. Meanwhile, the Korean Society for Bone and Mineral Research has opted for diagnosis using lower BMD scores out of the femoral neck and entire femur for femoral BMD.

The current study results demonstrate marked statistical associations between the lumbar spine and the three investigated liver enzyme levels. The spine shows a high proportion of trabecular bone which sensitively reflects change in bone metabolism in postmenopausal women. More consideration should be given to the fact that measurement errors in lumbar BMD measurements are high despite degenerative change occurring frequently in 65+ age groups [60], and further studies on the lumbar spine BMD are required. Also, DEXA is commonly used as the most sensitive and appropriate method for BMD assessment [61, 62], and future large-scale population studies may consider using DEXA as the standard for BMD measurement to heighten reliability. Multiple regression analysis was conducted to assess the association between liver enzymes and BMD using multivariate adjustment which enabled clearer relationships to be drawn. Although the association between liver enzyme levels and BMD did not necessarily pertain to associations between bone frailty or osteoporosis, the results hold significance in that they illustrate the trend between BMD and liver enzymes in a national sample.

## Conclusions

This study further clarifies the association between liver enzymes and BMD reported in previous U.S. studies. GGT and ALT exhibited negative associations with BMD at various sites, and out of BMD measurement regions, the lumbar spine displayed the most sensitive relationship with liver enzyme levels. As a large-scale study conducted on the relationship between liver enzymes and BMD in a homogeneous group, these findings support previous studies maintaining associations between osteoporosis and low bone density in hepatic disorders. Further detailed studies examining liver enzymes as potential risk factors of osteoporosis and low bone density are warranted.

## Additional file

Additional file 1: **Table S1–S3**. Association between liver function tests and whole femur and whole body bone mineral density as assessed using regression analysis models; association between liver function tests and femur neck and lumbar bone mineral density as assessed using regression analysis models; and subgroup analysis stratified by sex and menopausal status, or sex and age using multivariable-adjusted regression models. (DOCX 74 kb)

## Abbreviations

ALP: Alkaline phosphatase
ALT: Alanine aminotransferase
ANOVA: Analysis of variance
AST: Aspartate aminotransferase
BMD: Bone mineral density
BMI: Body mass index
CI: Confidence interval
DEXA: Dual energy X-ray absorptiometry
GGT: Gamma(γ)-glutamyl transferase
HIRA: Health Insurance Review and Assessment Service
IQR: Interquartile range
KNHANES: Korean National Health and Nutrition Examination Survey
NAFLD: nonalcoholic fatty liver disease
NHANES: National Health and Nutrition Examination Survey
WHO: World Health Organization
