# Supplementary material for: Association between liver enzymes and bone mineral density in Koreans: a cross-sectional study
Source: BMC Musculoskelet Disord. 2018 Nov 24;19:410. doi: 10.1186/s12891-018-2322-1 (PMC6260874; doi:10.1186/s12891-018-2322-1)
Supplement: Supplementary file 1 — Table S1–S3. Association between liver function tests and whole femur and whole body bone mineral density as assessed using regression analysis models; association between liver function tests and femur neck and lumbar bone mineral density as assessed using regression analysis models; and subgroup analysis stratified by sex and menopausal status, or sex and age using multivariable-adjusted regression models. (DOCX 74 kb) [file 12891_2018_2322_MOESM1_ESM.docx]

**Table S1** Association between liver function tests and whole femur and whole body bone mineral density as assessed using regression analysis models^a^

|  | Model 1 | | |  | Model 2 | | |  | Fully adjusted model | | |
| --- | --- | --- | --- | --- | --- | --- | --- | --- | --- | --- | --- |
| Whole femur | β | 95% CI | p-value |  | β | 95% CI | p-value |  | β | 95% CI | p-value |
| AST (IU/L) |  |  |  |  |  |  |  |  |  |  |  |
| AST >23 | 0.0229 | (0.0130,0.0328) | <.0001 |  | -0.0004 | (-0.0097,0.0089) | 0.9328 |  | 0.0007 | (-0.0086,0.0100) | 0.8819 |
| 19< AST ≤23 | 0.0148 | (0.0048,0.0247) | 0.0037 |  | 0.0010 | (-0.0078,0.0099) | 0.8173 |  | 0.0018 | (-0.0071,0.0106) | 0.6954 |
| 16< AST ≤19 | 0.0023 | (-0.0081,0.0126) | 0.6681 |  | -0.0026 | (-0.0116,0.0064) | 0.5715 |  | -0.0023 | (-0.0113,0.0067) | 0.6133 |
| AST ≤16 | Ref. |  |  |  | Ref. |  |  |  | Ref. |  |  |
| Adjusted R-Square |  |  | 0.3023 |  |  |  | 0.4071 |  |  |  | 0.4169 |
| per log unit |  |  | <.0001 |  |  |  | 0.7702 |  |  |  | 0.5784 |
| ALT (IU/L) |  |  |  |  |  |  |  |  |  |  |  |
| ALT >23 | 0.0545 | (0.0452,0.0637) | <.0001 |  | 0.0048 | (-0.0049,0.0146) | 0.3316 |  | 0.0052 | (-0.0047,0.0151) | 0.3013 |
| 16< ALT ≤23 | 0.0321 | (0.0218,0.0424) | <.0001 |  | 0.0034 | (-0.0061,0.0128) | 0.4825 |  | 0.0040 | (-0.0055,0.0135) | 0.4083 |
| 12< ALT ≤16 | 0.0228 | (0.0132,0.0323) | <.0001 |  | 0.0051 | (-0.0034,0.0137) | 0.2362 |  | 0.0056 | (-0.0029,0.0141) | 0.1969 |
| ALT ≤12 | Ref. |  |  |  | Ref. |  |  |  | Ref. |  |  |
| Adjusted R-Square |  |  | 0.3156 |  |  |  | 0.4072 |  |  |  | 0.4170 |
| per log unit |  |  | <.0001 |  |  |  | 0.9401 |  |  |  | 0.9261 |
| GGT (IU/L) |  |  |  |  |  |  |  |  |  |  |  |
| GGT >32 | 0.0361 | (0.0254,0.0468) | <.0001 |  | -0.0139 | (-0.0251,-0.0026) | 0.0157 |  | -0.0125 | (-0.024,-0.0011) | 0.0319 |
| 19< GGT ≤32 | 0.0308 | (0.0202,0.0413) | <.0001 |  | -0.0037 | (-0.0137,0.0063) | 0.4624 |  | -0.0023 | (-0.0123,0.0076) | 0.6458 |
| 13< GGT ≤19 | 0.0128 | (0.0029,0.0227) | 0.0113 |  | -0.0026 | (-0.0116,0.0063) | 0.5664 |  | -0.0016 | (-0.0103,0.0071) | 0.7145 |
| GGT ≤13 | Ref. |  |  |  | Ref. |  |  |  | Ref. |  |  |
| Adjusted R-Square |  |  | 0.3056 |  |  |  | 0.4081 |  |  |  | 0.4177 |
| per log unit |  |  | <.0001 |  |  |  | 0.0085 |  |  |  | 0.0202 |
|  | Model 1 | | |  | Model 2 | | |  | Fully adjusted model | | |
| Whole body | β | 95% CI | p-value |  | β | 95% CI | p-value |  | β | 95% CI | p-value |
| AST (IU/L) |  |  |  |  |  |  |  |  |  |  |  |
| AST >23 | 0.0055 | (-0.0042,0.0151) | 0.2664 |  | -0.0067 | (-0.0163,0.0029) | 0.1705 |  | -0.0048 | (-0.0145,0.0050) | 0.3352 |
| 19< AST ≤23 | 0.0049 | (-0.0044,0.0141) | 0.3009 |  | -0.0020 | (-0.0111,0.0070) | 0.6556 |  | -0.0007 | (-0.0096,0.0083) | 0.8843 |
| 16< AST ≤19 | -0.0017 | (-0.0108,0.0075) | 0.7234 |  | -0.0039 | (-0.0128,0.0049) | 0.3816 |  | -0.0029 | (-0.0117,0.0058) | 0.511 |
| AST ≤16 | Ref. |  |  |  | Ref. |  |  |  | Ref. |  |  |
| Adjusted R-Square |  |  | 0.2120 |  |  |  | 0.2472 |  |  |  | 0.2609 |
| per log unit |  |  | 0.0580 |  |  |  | 0.2773 |  |  |  | 0.4809 |
| ALT (IU/L) |  |  |  |  |  |  |  |  |  |  |  |
| ALT >23 | 0.0159 | (0.0065,0.0253) | 0.0010 |  | -0.0102 | (-0.0204,-0.0001) | 0.0488 |  | -0.0101 | (-0.0205,0.0004) | 0.0586 |
| 16< ALT ≤23 | 0.0064 | (-0.0027,0.0156) | 0.1684 |  | -0.0087 | (-0.0179,0.0005) | 0.0639 |  | -0.0065 | (-0.0158,0.0029) | 0.1734 |
| 12< ALT ≤16 | -0.0007 | (-0.0093,0.0079) | 0.8659 |  | -0.0102 | (-0.0185,-0.0019) | 0.0157 |  | -0.0088 | (-0.0170,-0.0006) | 0.0349 |
| ALT ≤12 | Ref. |  |  |  | Ref. |  |  |  | Ref. |  |  |
| Adjusted R-Square |  |  | 0.2141 |  |  |  | 0.2478 |  |  |  | 0.2615 |
| per log unit |  |  | 0.0002 |  |  |  | 0.0692 |  |  |  | 0.0769 |
| GGT (IU/L) |  |  |  |  |  |  |  |  |  |  |  |
| GGT >32 | 0.0080 | (-0.0033,0.0194) | 0.1643 |  | -0.0209 | (-0.0325,-0.0093) | 0.0005 |  | -0.0194 | (-0.0313,-0.0075) | 0.0015 |
| 19< GGT ≤32 | 0.0102 | (-0.0006,0.0210) | 0.0638 |  | -0.0096 | (-0.0203,0.0012) | 0.0802 |  | -0.0074 | (-0.0180,0.0033) | 0.1740 |
| 13< GGT ≤19 | -0.0009 | (-0.0107,0.0088) | 0.8525 |  | -0.0100 | (-0.0194,-0.0007) | 0.0361 |  | -0.0080 | (-0.0171,0.0012) | 0.0866 |
| GGT ≤13 | Ref. |  |  |  | Ref. |  |  |  | Ref. |  |  |
| Adjusted R-Square |  |  | 0.2127 |  |  |  | 0.2491 |  |  |  | 0.2627 |
| per log unit |  |  | 0.2154 |  |  |  | 0.0013 |  |  |  | 0.0031 |

^a^ Model 1: adjusted for age, and sex;

Model 2: adjusted for age, sex, body mass index (BMI), and alcohol use;

Fully adjusted model: adjusted for age, sex, BMI, alcohol use, smoking status, diabetes, physical activity, and education

Age and BMI are adjusted as continuous variables.

AST, aspartate aminotransferase; ALT, alanine aminotransferase; GGT, γ-glutamyl transferase

**Table S2** Association between liver function tests and femur neck and lumbar bone mineral density as assessed using regression analysis models^a^

|  |  | Model 1 | | |  | Model 1-a | | |  | Model 1-b | | |  | Model 2 | | |  | Fully adjusted model | | |
| --- | --- | --- | --- | --- | --- | --- | --- | --- | --- | --- | --- | --- | --- | --- | --- | --- | --- | --- | --- | --- |
| Femur neck | | β | 95% CI | p-value |  | β | 95% CI | p-value |  | β | 95% CI | p-value |  | β | 95% CI | p-value |  | β | 95% CI | p-value |
| AST (IU/L) | |  |  |  |  |  |  |  |  |  |  |  |  |  |  |  |  |  |  |  |
|  | AST >23 | 0.0179 | (0.0081,0.0277) | 0.0004 |  | 0.0169 | (0.0071,0.0266) | 0.0008 |  | 0.0011 | (-0.0086,0.0107) | 0.8305 |  | 0.0001 | (-0.0096,0.0097) | 0.9893 |  | 0.0008 | (-0.009,0.0105) | 0.8791 |
|  | 19< AST ≤23 | 0.0101 | (0.0006,0.0196) | 0.0366 |  | 0.0097 | (0.0003,0.0192) | 0.0427 |  | 0.0009 | (-0.0082,0.0099) | 0.853 |  | 0.0005 | (-0.0085,0.0094) | 0.9177 |  | 0.0006 | (-0.0083,0.0095) | 0.8924 |
|  | 16< AST ≤19 | 0.0016 | (-0.0085,0.0118) | 0.7518 |  | 0.0015 | (-0.0086,0.0115) | 0.7748 |  | -0.0008 | (-0.0102,0.0086) | 0.8704 |  | -0.0009 | (-0.0103,0.0084) | 0.8431 |  | -0.0010 | (-0.0103,0.0083) | 0.8296 |
|  | AST ≤16 | Ref. |  |  |  | Ref. |  |  |  | Ref. |  |  |  | Ref. |  |  |  | Ref. |  |  |
|  | per log unit |  |  | 0.0002 |  |  |  | 0.0005 |  |  |  | -0.19 |  |  |  | 0.6985 |  |  |  | 0.8629 |
|  | age*male | 0.0000 | (-0.0004,0.0005) | 0.8278 |  | 0.0000 | (-0.0005,0.0004) | 0.9756 |  | 0.0007 | (0.0003,0.0012) | 0.0015 |  | 0.0007 | (0.0002,0.0011) | 0.0034 |  | 0.0007 | (0.0002,0.0011) | 0.0066 |
|  | age*female |  |  |  |  |  |  |  |  |  |  |  |  |  |  |  |  |  |  |  |
|  | Adjusted R-Square | 0.37 | | |  | 0.37 | | |  | 0.43 | | |  | 0.43 | | |  | 0.44 | | |
| ALT (IU/L) | |  |  |  |  |  |  |  |  |  |  |  |  |  |  |  |  |  |  |  |
|  | ALT >23 | 0.0392 | (0.0301,0.0484) | <.0001 |  | 0.0391 | (0.0299,0.0482) | <.0001 |  | -0.0008 | (-0.0111,0.0095) | 0.8818 |  | -0.0009 | (-0.0112,0.0095) | 0.8666 |  | 0.0003 | (-0.0102,0.0108) | 0.9578 |
|  | 16< ALT ≤23 | 0.0239 | (0.0142,0.0336) | <.0001 |  | 0.0240 | (0.0143,0.0336) | <.0001 |  | 0.0012 | (-0.0081,0.0106) | 0.7947 |  | 0.0013 | (-0.008,0.0106) | 0.7826 |  | 0.0015 | (-0.0078,0.0108) | 0.7506 |
|  | 12< ALT ≤16 | 0.0173 | (0.0092,0.0253) | <.0001 |  | 0.0170 | (0.009,0.025) | <.0001 |  | 0.0042 | (-0.0035,0.0119) | 0.2863 |  | 0.0040 | (-0.0038,0.0117) | 0.3136 |  | 0.0037 | (-0.0041,0.0114) | 0.3504 |
|  | ALT ≤12 | Ref. |  |  |  | Ref. |  |  |  | Ref. |  |  |  | Ref. |  |  |  | Ref. |  |  |
|  | per log unit |  |  | <.0001 |  |  |  | <.0001 |  |  |  | 0.3045 |  |  |  | 0.3093 |  |  |  | 0.4769 |
|  | age*male | 0.0002 | (-0.0002,0.0007) | 0.2916 |  | 0.0002 | (-0.0003,0.0006) | 0.424 |  | 0.0008 | (0.0003,0.0012) | 0.0012 |  | 0.0007 | (0.0002,0.0012) | 0.0028 |  | 0.0007 | (0.0002,0.0011) | 0.006 |
|  | age*female |  |  |  |  |  |  |  |  |  |  |  |  |  |  |  |  |  |  |  |
|  | Adjusted R-Square | 0.37 | | |  | 0.37 | | |  | 0.43 | | |  | 0.43 | | |  | 0.44 | | |
| GGT (IU/L) | |  |  |  |  |  |  |  |  |  |  |  |  |  |  |  |  |  |  |  |
|  | GGT >32 | 0.0209 | (0.0107,0.0312) | <.0001 |  | 0.0177 | (0.0073,0.0281) | 0.0009 |  | -0.0155 | (-0.0258,-0.0051) | 0.0036 |  | -0.0201 | (-0.0307,-0.0094) | 0.0003 |  | -0.0186 | (-0.0295,-0.0077) | 0.0009 |
|  | 19< GGT ≤32 | 0.0199 | (0.0101,0.0296) | <.0001 |  | 0.0181 | (0.0083,0.0278) | 0.0003 |  | -0.0048 | (-0.0143,0.0047) | 0.3188 |  | -0.0074 | (-0.017,0.0021) | 0.1257 |  | -0.0066 | (-0.0162,0.003) | 0.1762 |
|  | 13< GGT ≤19 | 0.0078 | (-0.0016,0.0173) | 0.1032 |  | 0.0070 | (-0.0024,0.0164) | 0.1425 |  | -0.0022 | (-0.0112,0.0069) | 0.6366 |  | -0.0034 | (-0.0123,0.0056) | 0.462 |  | -0.0033 | (-0.012,0.0055) | 0.4636 |
|  | GGT ≤13 | Ref. |  |  |  | Ref. |  |  |  | Ref. |  |  |  | Ref. |  |  |  | Ref. |  |  |
|  | per log unit |  |  | 0.0029 |  |  |  | 0.0234 |  |  |  | 0.001 |  |  |  | <.0001 |  |  |  | 0.0003 |
|  | age*male | 0.0001 | (-0.0004,0.0005) | 0.7571 |  | 0.0000 | (-0.0004,0.0005) | 0.9428 |  | 0.0008 | (0.0003,0.0012) | 0.0011 |  | 0.0007 | (0.0002,0.0012) | 0.003 |  | 0.0007 | (0.0002,0.0011) | 0.0069 |
|  | age*female |  |  |  |  |  |  |  |  |  |  |  |  |  |  |  |  |  |  |  |
|  | Adjusted R-Square | 0.37 | | |  | 0.37 | | |  | 0.43 | | |  | 0.44 | | |  | 0.44 | | |
|  |  | Model 1 | | |  | Model 1-a | | |  | Model 1-b | | |  | Model 2 | | |  | Fully adjusted model | | |
| Lumbar spine | | β | 95% CI | p-value |  | β | 95% CI | p-value |  | β | 95% CI | p-value |  | β | 95% CI | p-value |  | β | 95% CI | p-value |
| AST (IU/L) | |  |  |  |  |  |  |  |  |  |  |  |  |  |  |  |  |  |  |  |
|  | AST >23 | 0.0027 | (-0.009,0.0143) | 0.6535 |  | 0.0019 | (-0.0097,0.0134) | 0.749 |  | -0.0137 | (-0.0253,-0.0021) | 0.0204 |  | -0.0146 | (-0.0261,-0.003) | 0.0137 |  | -0.0148 | (-0.0263,-0.0032) | 0.0123 |
|  | 19< AST ≤23 | -0.0040 | (-0.0153,0.0074) | 0.4918 |  | -0.0043 | (-0.0156,0.007) | 0.4563 |  | -0.0123 | (-0.023,-0.0016) | 0.0245 |  | -0.0126 | (-0.0232,-0.002) | 0.0201 |  | -0.0150 | (-0.0255,-0.0046) | 0.0049 |
|  | 16< AST ≤19 | -0.0066 | (-0.0177,0.0045) | 0.2412 |  | -0.0067 | (-0.0178,0.0043) | 0.2314 |  | -0.0084 | (-0.019,0.0022) | 0.1206 |  | -0.0085 | (-0.0191,0.0021) | 0.1142 |  | -0.0106 | (-0.0209,-0.0002) | 0.0457 |
|  | AST ≤16 | Ref. |  |  |  | Ref. |  |  |  | Ref. |  |  |  | Ref. |  |  |  | Ref. |  |  |
|  | age*male | 0.0029 | (0.0024,0.0035) | <.0001 |  | 0.0029 | (0.0023,0.0035) | <.0001 |  | 0.0037 | (0.0031,0.0042) | <.0001 |  | 0.0036 | (0.0031,0.0042) | <.0001 |  | 0.0034 | (0.0028,0.004) | <.0001 |
|  | age*female |  |  |  |  |  |  |  |  |  |  |  |  |  |  |  |  |  |  |  |
|  | Adjusted R-Square | 0.14 | | |  | 0.14 | | |  | 0.21 | | |  | 0.21 | | |  | 0.24 | | |
|  | per log unit |  |  | 0.6864 |  |  |  | 0.8006 |  |  |  | 0.0042 |  |  |  | 0.0027 |  |  |  | 0.0067 |
| ALT (IU/L) | |  |  |  |  |  |  |  |  |  |  |  |  |  |  |  |  |  |  |  |
|  | ALT >23 | 0.0331 | (0.0211,0.045) | <.0001 |  | 0.0331 | (0.0211,0.045) | <.0001 |  | -0.0085 | (-0.0216,0.0046) | 0.2041 |  | -0.0085 | (-0.0216,0.0046) | 0.2035 |  | -0.0160 | (-0.0294,-0.0026) | 0.0193 |
|  | 16< ALT ≤23 | 0.0210 | (0.0105,0.0315) | 0.0001 |  | 0.0211 | (0.0106,0.0316) | <.0001 |  | -0.0023 | (-0.013,0.0083) | 0.6658 |  | -0.0022 | (-0.0129,0.0085) | 0.6855 |  | -0.0083 | (-0.019,0.0025) | 0.1312 |
|  | 12< ALT ≤16 | 0.0103 | (0.0009,0.0197) | 0.0319 |  | 0.0101 | (0.0007,0.0195) | 0.0345 |  | -0.0037 | (-0.0127,0.0053) | 0.4236 |  | -0.0038 | (-0.0128,0.0052) | 0.4014 |  | -0.0080 | (-0.0169,0.0009) | 0.0765 |
|  | ALT ≤12 | Ref. |  |  |  | Ref. |  |  |  | Ref. |  |  |  | Ref. |  |  |  | Ref. |  |  |
|  | age*male | 0.0032 | (0.0026,0.0037) | <.0001 |  | 0.0031 | (0.0026,0.0037) | <.0001 |  | 0.0037 | (0.0032,0.0043) | <.0001 |  | 0.0037 | (0.0031,0.0042) | <.0001 |  | 0.0034 | (0.0028,0.004) | <.0001 |
|  | age*female |  |  |  |  |  |  |  |  |  |  |  |  |  |  |  |  |  |  |  |
|  | Adjusted R-Square | 0.15 | | |  | 0.15 | | |  | 0.21 | | |  | 0.21 | | |  | 0.24 | | |
|  | per log unit |  |  | <.0001 |  |  |  | <.0001 |  |  |  | 0.0701 |  |  |  | 0.072 |  |  |  | 0.0601 |
| GGT (IU/L) | |  |  |  |  |  |  |  |  |  |  |  |  |  |  |  |  |  |  |  |
|  | GGT >32 | 0.0167 | (0.0033,0.0301) | 0.0149 |  | 0.0143 | (0.0006,0.028) | 0.0409 |  | -0.0205 | (-0.0341,-0.0069) | 0.0032 |  | -0.0245 | (-0.0384,-0.0105) | 0.0006 |  | -0.0273 | (-0.0412,-0.0133) | 0.0002 |
|  | 19< GGT ≤32 | 0.0190 | (0.0071,0.0309) | 0.0019 |  | 0.0177 | (0.0057,0.0298) | 0.0041 |  | -0.0054 | (-0.017,0.0062) | 0.3615 |  | -0.0076 | (-0.0193,0.0042) | 0.2049 |  | -0.0082 | (-0.0195,0.0031) | 0.1518 |
|  | 13< GGT ≤19 | 0.0020 | (-0.0098,0.0138) | 0.7381 |  | 0.0015 | (-0.0103,0.0133) | 0.8051 |  | -0.0082 | (-0.0191,0.0026) | 0.137 |  | -0.0091 | (-0.02,0.0018) | 0.1002 |  | -0.0082 | (-0.0184,0.0021) | 0.1171 |
|  | GGT ≤13 | Ref. |  |  |  | Ref. |  |  |  | Ref. |  |  |  | Ref. |  |  |  | Ref. |  |  |
|  | age*male | 0.0030 | (0.0025,0.0036) | <.0001 |  | 0.0030 | (0.0024,0.0036) | <.0001 |  | 0.0037 | (0.0032,0.0043) | <.0001 |  | 0.0037 | (0.0031,0.0042) | <.0001 |  | 0.0035 | (0.0029,0.004) | <.0001 |
|  | age*female |  |  |  |  |  |  |  |  |  |  |  |  |  |  |  |  |  |  |  |
|  | Adjusted R-Square | 0.14 | | |  | 0.14 | | |  | 0.21 | | |  | 0.21 | | |  | 0.24 | | |
|  | per log unit |  |  | 0.166 |  |  |  | 0.3319 |  |  |  | 0.0012 |  |  |  | 0.0002 |  |  |  | 0.0008 |

^a^ Model 1: adjusted for age, sex, and age*sex;

Model 1-a: adjusted for age, sex, alcohol use, and age*sex;

Model 1-b: adjusted for age, sex, body mass index (BMI), and age*sex;

Model 2: adjusted for age, sex, alcohol use, BMI, and age*sex;

Fully adjusted model: adjusted for age, sex, alcohol use, BMI, smoking status, diabetes, physical activity, education, and age*sex

**Table S3** Subgroup analysis stratified by sex and menopausal status, or sex and age using multivariable-adjusted regression model^a^

|  |  | Premenopause | | |  | Postmenopause | | |  | Males aged 41 and under | | |  | Males aged over 41 | | |
| --- | --- | --- | --- | --- | --- | --- | --- | --- | --- | --- | --- | --- | --- | --- | --- | --- |
| Femur neck |  | β | 95% CI | p-value |  | β | 95% CI | p-value |  | β | 95% CI | p-value |  | β | 95% CI | p-value |
| AST (IU/L) |  |  |  |  |  |  |  |  |  |  |  |  |  |  |  |  |
| AST >23 |  | 0.5795 | (0.5399,0.6191) | <.0001 |  | 0.0029 | (-0.0142,0.02) | 0.7378 |  | -0.0029 | (-0.027,0.0212) | 0.8146 |  | 0.0069 | (-0.0158,0.0296) | 0.5509 |
| 19< AST ≤23 |  | 0.0225 | (0.0013,0.0437) | 0.0376 |  | 0.0058 | (-0.0118,0.0234) | 0.5174 |  | 0.0066 | (-0.0168,0.03) | 0.5778 |  | 0.0095 | (-0.014,0.033) | 0.4245 |
| 16< AST ≤19 |  | -0.0109 | (-0.0243,0.0026) | 0.1142 |  | 0.0036 | (-0.0155,0.0227) | 0.7089 |  | 0.0041 | (-0.0196,0.0278) | 0.7318 |  | -0.0002 | (-0.0257,0.0253) | 0.9886 |
| AST ≤16 |  | Ref. |  |  |  | Ref. |  |  |  | Ref. |  |  |  | Ref. |  |  |
| per log unit |  |  |  | 0.5596 |  |  |  | 0.4681 |  |  |  | 0.5403 |  |  |  | 0.3652 |
| ALT (IU/L) |  |  |  |  |  |  |  |  |  |  |  |  |  |  |  |  |
| ALT >23 |  | 0.0063 | (-0.0151,0.0277) | 0.5620 |  | -0.0126 | (-0.0279,0.0027) | 0.1061 |  | 0.0120 | (-0.0179,0.0419) | 0.4295 |  | 0.0053 | (-0.015,0.0257) | 0.6073 |
| 16< ALT ≤23 |  | -0.0114 | (-0.026,0.0032) | 0.1247 |  | 0.0002 | (-0.015,0.0155) | 0.9767 |  | 0.0056 | (-0.0233,0.0345) | 0.7023 |  | 0.0092 | (-0.011,0.0295) | 0.3693 |
| 12< ALT ≤16 |  | -0.0013 | (-0.0128,0.0101) | 0.8198 |  | -0.0092 | (-0.0233,0.0049) | 0.1995 |  | 0.0175 | (-0.0108,0.0458) | 0.2234 |  | 0.0071 | (-0.0147,0.0289) | 0.5204 |
| ALT ≤12 |  | Ref. |  |  |  | Ref. |  |  |  | Ref. |  |  |  | Ref. |  |  |
| per log unit |  |  |  | 0.6873 |  |  |  | 0.1684 |  |  |  | 0.7985 |  |  |  | 0.3967 |
| GGT (IU/L) |  |  |  |  |  |  |  |  |  |  |  |  |  |  |  |  |
| GGT >32 |  | -0.0174 | (-0.0378,0.0029) | 0.0927 |  | -0.0198 | (-0.0363,-0.0032) | 0.0195 |  | -0.0183 | (-0.0564,0.0199) | 0.3465 |  | 0.0064 | (-0.0282,0.041) | 0.7152 |
| 19< GGT ≤32 |  | -0.0089 | (-0.0235,0.0056) | 0.2286 |  | -0.0192 | (-0.0343,-0.0041) | 0.0127 |  | -0.0018 | (-0.0419,0.0383) | 0.9300 |  | 0.0165 | (-0.0187,0.0516) | 0.3569 |
| 13< GGT ≤19 |  | -0.0013 | (-0.0114,0.0088) | 0.8000 |  | -0.0158 | (-0.029,-0.0026) | 0.0194 |  | 0.0022 | (-0.0348,0.0391) | 0.9074 |  | 0.0035 | (-0.0316,0.0385) | 0.8452 |
| GGT ≤13 |  | Ref. |  |  |  | Ref. |  |  |  | Ref. |  |  |  | Ref. |  |  |
| per log unit |  |  |  | 0.4401 |  |  |  | 0.0317 |  |  |  | 0.2613 |  |  |  | 0.0401 |
|  |  | Premenopause | | |  | Postmenopause | | |  | Males aged 41 and under | | |  | Males aged over 41 | | |
| Lumbar spine |  | β | 95% CI | p-value |  | β | 95% CI | p-value |  | β | 95% CI | p-value |  | β | 95% CI | p-value |
| AST (IU/L) |  |  |  |  |  |  |  |  |  |  |  |  |  |  |  |  |
| AST >23 |  | 0.6966 | (0.652,0.7412) | <.0001 |  | 0.0006 | (-0.0257,0.027) | 0.9618 |  | -0.0091 | (-0.0366,0.0184) | 0.5153 |  | -0.0071 | (-0.0356,0.0214) | 0.6221 |
| 19< AST ≤23 |  | 0.0014 | (-0.0222,0.0249) | 0.9099 |  | -0.0071 | (-0.0332,0.019) | 0.5919 |  | -0.0185 | (-0.043,0.0061) | 0.1399 |  | 0.0070 | (-0.0221,0.0361) | 0.6367 |
| 16< AST ≤19 |  | -0.0131 | (-0.0301,0.0039) | 0.1309 |  | -0.0020 | (-0.0292,0.0251) | 0.8830 |  | -0.0133 | (-0.0369,0.0102) | 0.2664 |  | -0.0123 | (-0.0495,0.0248) | 0.5142 |
| AST ≤16 |  | Ref. |  |  |  | Ref. |  |  |  | Ref. |  |  |  | Ref. |  |  |
| per log unit |  |  |  | 0.2530 |  |  |  | 0.7763 |  |  |  | 0.7485 |  |  |  | 0.0383 |
| ALT (IU/L) |  |  |  |  |  |  |  |  |  |  |  |  |  |  |  |  |
| ALT >23 |  | -0.0021 | (-0.0253,0.0211) | 0.8592 |  | -0.0018 | (-0.0272,0.0235) | 0.8861 |  | 0.0104 | (-0.0204,0.0413) | 0.5058 |  | -0.0123 | (-0.0454,0.0208) | 0.4633 |
| 16< ALT ≤23 |  | -0.0019 | (-0.0181,0.0143) | 0.8177 |  | -0.0009 | (-0.0219,0.0201) | 0.9315 |  | 0.0143 | (-0.0131,0.0417) | 0.3064 |  | -0.0040 | (-0.0364,0.0283) | 0.8066 |
| 12< ALT ≤16 |  | -0.0057 | (-0.0183,0.0068) | 0.3691 |  | -0.0060 | (-0.028,0.0159) | 0.5899 |  | 0.0051 | (-0.0242,0.0344) | 0.7310 |  | -0.0090 | (-0.0429,0.0249) | 0.6017 |
| ALT ≤12 |  | Ref. |  |  |  | Ref. |  |  |  | Ref. |  |  |  | Ref. |  |  |
| per log unit |  |  |  | 0.6052 |  |  |  | 0.8292 |  |  |  | 0.6968 |  |  |  | 0.3601 |
| GGT (IU/L) |  |  |  |  |  |  |  |  |  |  |  |  |  |  |  |  |
| GGT >32 |  | -0.0077 | (-0.0344,0.019) | 0.5709 |  | -0.0170 | (-0.0461,0.0121) | 0.2520 |  | -0.0193 | (-0.054,0.0153) | 0.2725 |  | 0.0091 | (-0.0328,0.051) | 0.6689 |
| 19< GGT ≤32 |  | -0.0162 | (-0.0328,0.0004) | 0.0554 |  | -0.0026 | (-0.0255,0.0203) | 0.8250 |  | 0.0031 | (-0.0318,0.038) | 0.8610 |  | 0.0213 | (-0.0219,0.0645) | 0.3323 |
| 13< GGT ≤19 |  | -0.0055 | (-0.0172,0.0062) | 0.3553 |  | -0.0239 | (-0.0449,-0.0029) | 0.0256 |  | -0.0017 | (-0.037,0.0336) | 0.9242 |  | 0.0155 | (-0.0283,0.0593) | 0.4864 |
| GGT ≤13 |  | Ref. |  |  |  | Ref. |  |  |  | Ref. |  |  |  | Ref. |  |  |
| per log unit |  |  |  | 0.5557 |  |  |  | 0.7728 |  |  |  | 0.1674 |  |  |  | 0.0120 |

^a^Adjusted for age, sex, body mass index (BMI), alcohol use, diabetes, physical activity, education, and smoking status

AST, aspartate aminotransferase; ALT, alanine aminotransferase; GGT, γ-glutamyl transferase
